# Food Safety Conditions in Home-Kitchens: A Cross-Sectional Study in the Federal District/Brazil

**DOI:** 10.3390/ijerph17134897

**Published:** 2020-07-07

**Authors:** Adenilma da Silva Farias, Rita de Cassia Coelho de Almeida Akutsu, Raquel Braz Assunção Botelho, Wilma Maria Coelho Araújo, Izabel Cristina Silva, Karin Eleonora Sávio, Renata Puppin Zandonadi

**Affiliations:** 1 Federal Institute of Piauí, Campus Pedro II, Piauí 64255-000, Brazil; adenilma.farias@ifpi.edu.br; 2 Department of Nutrition, Faculty of Health Sciences, University of Brasilia, Brasilia 70910-900, Brazil; raquelbabotelho@gmail.com (R.B.A.B.); wilma.araujo@terra.com.br (W.M.C.A.); kesavio@unb.br (K.E.S.); renatapz@yahoo.com.br (R.P.Z.); 3 Faculty of Ceilândia, University of Brasília, Brasília 72220-275, Brazil; belbiomedica@gmail.com

**Keywords:** foodborne diseases, home-kitchens, good habits, prevention, instrument, reliability

## Abstract

This study aimed to analyze the food safety conditions in home kitchens from the Brazilian Federal District. A previously validated instrument composed of 77 items (in four blocks) was used to evaluate the safety conditions in home kitchens. A survey was carried out with on-site application with 226 home kitchens’ food handlers in the Federal District, Brazil to evaluate Brazilian home kitchens’ good practices. Of the home kitchen food handlers, most of them were female (64.6%), had completed undergraduate education (29.2%), and were 45–59 years old (23.5%). The visited households had an average of 3.38 ± 1.48 residents that ate some of their meals at home, and 40% declared the monthly family income to be between 5 and 15 Brazilian minimum wages (MW). Regarding the reliability of the instrument, from the KR-20 test, it was verified that the instrument presents good internal consistency (α = 0.758). According to the instrument classification, the home kitchens’ sample was considered as at a medium risk of food contamination (46.5% of them presented from 51 to 75% of conformities to the instrument). There was a statistical difference between house-kitchens with a family income from zero to one minimum wage (MW) and those receiving from 5 to 15 MW (*p* = 0.017), as well as between those from zero to one MW and who earn above 15 MW (*p* = 0.009). The result of the on-site evaluation shows that the instrument was able to measure food safety conditions in Brazilian Federal District domestic kitchens. Such findings can contribute positively to the development of actions in health education that help in the adoption of good practices of food manipulation and, consequently, in the reduction in foodborne disease outbreaks in residences.

## 1. Introduction

Foodborne diseases (FBDs) comprise a broad spectrum of illnesses that result from the ingestion of contaminated foodstuffs or water. FBDs can occur at any stage, from production to food consumption, and contamination may be the result of environmental contamination, such as pollution of water, soil, or air [1]. FBDs represent one of the most critical public health problems in the world, especially in developing countries that present serious shortcomings in infrastructure and basic sanitation [2,3,4,5]. Although they represent a threat to public health and hamper global socioeconomic development, FBDs are not among the priorities for public health prevention and care, especially in developing countries [6]. In general, the countries presenting the highest occurrence rates are those that have the fewest resources to prevent them [2,3].

In Brazil, between 2007 and 2018, 134,046 cases of FBDs were recorded, with 19,394 hospitalizations and 129 deaths [4]. Another recent study [5] assessed the FBD outbreaks reported in Brazil between 2000 and 2018, based on the Brazilian official data and the scientific literature. It showed that 13,163 FBD outbreaks were reported in the country during this period, involving 247,570 cases and 195 deaths. Both Brazilian studies highlighted the home as the primary site of FBD occurrence (38.3% [4], and 12.5% [5]). It is essential to consider that reports showed that most cases of FBD are not notified to health authorities; because many foodborne pathogens cause mild symptoms, victims do not seek medical help, contributing to the underreporting of epidemiological data [4,5].

Outbreaks of FBD from homemade foods are mainly a consequence of the lack of education on health and food safety of the general population, especially a knowledge gap about the adequate preparation and storage of food by the population in general [7,8]. Although to a lesser extent, it can also be a consequence of the consumption of foods contaminated at the retail level. For instance, consumers might use a contaminated ingredient in a recipe prepared at home. In such cases, the cause of the FBD might be contamination that has happened before the food preparation at home. It is also important to mention that home ingestion does not mean that the foods were prepared at home. Supermarkets in Brazil sell ready-to-eat foods, and there are delivery services via apps or phones that are popular in Brazil. In certain circumstances, meals can have foods partially prepared at home or a mix of homemade dishes and foods prepared by restaurants.

Outbreaks of FBD in households tend to be less well-known because they involve a smaller number of people (usually family). Consumers/food handlers do not see themselves or a family member as a contamination source or as susceptible to the risk of FBD [9]. These facts contribute to the absence of direction in educational campaigns and training for the public [4,10,11].

Improving consumers’ knowledge, attitudes, and behaviors concerning food safety can be important to the prevention of FBD because the population has difficulties perceiving the risk in domestic food preparation and consumption [12,13]. It is necessary to know the main FBD risk attitudes regarding the preparation and consumption of food at home to create communication and strategies for educational interventions in food safety [12]. It can potentially reduce the FBD cases if people adopt preventive actions throughout home food preparation and consumption. Despite the concerns about the home’s FBDs, research about food safety conditions in home-kitchens is still scarce [14,15]. According to the World Health Organization, 30 to 40% of FBDs occur at home [16]. The Institute of Health in Italy pointed out that 55 to 75% of all outbreaks occur inside households [17]. In Brazil, the government regulates food safety practices in commercial food services, but it is not the responsibility of the government to control food practices at home. However, education on food safety at home should be included as part of public policies from an early age. Additionally, health professionals should include counseling on food safety as part of their educational process with clients. Considering the importance of the knowledge to prevent FBDs, and the concerns about the increase in FBD cases arising in households, it is essential to evaluate homes’ good food preparation practices. Therefore, this study aimed to evaluate the food safety conditions in home kitchens from the Brazilian Federal District, potentially allowing future interventions and designing public policies to prevent FBDs and reduce public health costs.

## 2. Materials and Methods 

This cross-sectional study was performed in the Brazilian Federal District using a previously validated questionnaire to evaluate Brazilian home kitchens’ good practices, composed of 77 items divided into four blocks (1. Construction and facilities; 2. Equipment, furniture, and kitchen utensils; 3. Handlers; and 4. Food and Feedstock) [18]. The questionnaire to evaluate home kitchens accepted three types of answer per item in each block (yes, no, or not applicable).

The first block (construction and facilities) was composed of 14 sections with 36 items (1.1 External Area: External area free of outbreaks of unhealthiness, garbage accumulation, stagnant water, among others; Access roads with an adequate sewage system. 1.2 Internal Area: Kitchen free of obsolete objects or strangers to the environment; Free of the presence of domestic animals. 1.3 Floor: In good state of cleanliness; Cleaned at least once a day; Floor in a material that facilitates its cleaning (ceramics or similar); In a suitable state of conservation (free of defects, cracks, cracks, holes, and others). 1.4 Kitchen Ceiling: With appropriate ceiling material that facilitates cleaning (plaster, PVC, concrete, or similar); In a suitable state of conservation (free of cracks, humidity, mold, fungus, spider webs, paint peel, and others). 1.5 Walls and Kitchen Divisions: In good state of cleanliness; Built with a material of easy cleansing; In a suitable state of conservation (free of cracks, humidity, paint peel, and others). 1.6 Kitchen Door: In a suitable state of conservation (free of cracks, humidity, paint peel, and others); Built with a material of easy cleansing. 1.7 Windows and Other Kitchen Openings: In a suitable state of conservation (free of cracks, humidity, paint peel, and others); Built with a material of easy cleansing. 1.8 Toilets: Toilet with an intact toilet seat and with lid; Toilet with running water and connected to the sewage system or septic tank; Toilet without direct link to the kitchen area and/or the dining room; Toilet with bins with lids and pedal triggering. 1.9 Lighting and kitchen electrical wiring: Recessed lighting or when external, covered by insulating pipes attached to walls and ceiling; Bulbs and electric switches free of dirt; Cleaning of lamps, outlets, and electric switches at least once a month. 1.10 Ventilation and Acclimatization System of the Kitchen: Kitchen with ventilation and air circulation capable of thermal comfort; Kitchen free of fungus, causing no harm to food; 1.11 Urban Vector and Pest Control: Absence of urban vectors and pests or other evidence such as feces, nests, and other; Disinfecting every six months. 1.12 Water Supply: Water supply system connected to the public grid; Proper, protected, covered, and distant from contamination water system; Water tank with lid and in a good state; Cleaning of water tank every six months. 1.13 Waste Management: Easy to clean and carry waste bins; in a proper state and with appropriate garbage bags; Covered waste bins with pedal triggering; No waste bins over the sink; Waste stored in appropriate areas. 1.14 Sewage System: Septic tank and sewage system connected to the public sewage).

The second block (Equipment, Furniture, and Kitchen Utensils) was composed of four sections with 25 items (2.1 Equipment: Fridge and stove in an area that permits adequate cleaning; Conservation equipment for food (fridges, freezers, and others) in proper functioning; Thermal food equipment (stove, oven, and/or microwave) in proper functioning; 2.2 Furniture (tables, countertop, cupboard, shelves): From resistant material with proper surface conditions; Withdrawing that allows easy cleaning (smooth, without roughness, and chinks). 2.3 Utensils: Size and shape for easy cleaning and in a proper state; No wooden utensils or other materials of easy contamination; Pans, pots, and trays in a proper state; Boards, knives, skimmers, and holders in a proper state; Slicers and squeezers in a proper state; Utensils (plates, silverware, bowls) in proper state. 2.4 Equipment, Furniture, and Kitchen Utensils Cleaning: Fridges and freezers in a proper state; Fridges or freezers cleaned at least once a week; The stove is cleaned when used; Dishcloths in a proper and cleaned state; Dishrags or table rags in a proper state; Dishcloths are changed daily; Dishrags are changed daily; Cleaning sponges in proper hygiene and state; Cleaning sponges changed weekly; Cleaning products approved by the health department; Cleaning products in their original packing and stored in proper location; Equipment, furniture, and kitchen utensils in proper hygiene and state; Water filters changed every six months; No sponges from steel or wool).

The third block (Handlers) was composed of two sections with three items (3.1 Hygiene Habits: Personal cleaning, good appearance, clean hands, short nails, and unadorned (rings, earrings, bracelets, others); Handlers with previous knowledge on hand washing. 3.2 Health Condition: Absence of skin rash, wound, suppuration, absence of respiratory, eye, and gastric infections). The last block (Food and feedstock) was composed of two sections with 13 items (4.1 Food and Feedstock Origin: Food and feedstock with labels and packaging according to legislation; Milk from a secure source; Cheese from a secure source, packed and labeled; Meat, chicken, or fish from proper establishments; Filtered or boiled water consumption. 4.2 Food Storage: Adequate Semi perishable food stored and organized area with air circulation and lighting; Food prepared in advance before serving heated again; Fridges and freezers organized in order to avoid cross-contamination; Feedstock not used are entirely correctly stored in a clean and closed container; Feedstock not used are entirely identified with expiration date; Perishable food stored in an adequate temperature; Packages well cleaned before used for a fridge or freeze storage; Leftovers stored under refrigeration and with bowls with lids.

The Brasilia University Research Ethics Committee approved the study on 6 June 2014 (CAAE: 23955313.3.0000.0030).

### 2.1. Sample Sizing and Application of Instrument

The questionnaire was applied in the home kitchens of the Brazilian Federal District (FD) in stratified sampling by social class. According to the last Federal District Household Survey-PDAD [19], the FD had 886,395 registered households in 2015. The sample size was initially defined by adopting a sampling error of 0.05 and a confidence level of 95% for normal distribution and a 50% population estimate, considering the heterogeneity of the population [20]. The registered households list includes homes from five regions of the FD. Therefore, houses were drawn from the registration list for each region to be contacted. If a house did not agree to participate, another house was drawn from the same region.

The minimum sample size to be representative of the Federal District was at least 213 households. Thus, 226 home kitchens were studied in five different regions of the FD. The eligibility criteria were: (i) the home participants must be FD residents and ≥18 years old; (ii) the interviewees should be food handlers in their homes; and (iii) individuals should consent to participate in the study, allowing the data collection in their homes. After completing the eligibility criteria and signing the consent form, the researcher entered each home kitchen to collect the data and fill in the previously validated questionnaire [18]. The research was conducted in Brazilian-Portuguese.

Evaluation of collected data occurred by classifying the home kitchen as low, medium, or high sanitary risk. The Brazilian legislation scale from RDC nº 275/02 for industries classifies establishments, dividing the results according to the percentage of conformity of the analyzed items (Group 1—Low risk—76 to 100% of attendance; Group 2—Medium risk—51 to 75% attendance; Group 3—High risk—0 to 50% attendance) [21]. We used the same strategy in this study.

### 2.2. Statistical Analysis

For the statistical analysis, we used the Statistical Package for the Social Sciences—SPSS 24.0 (version 24, SPSS Inc., Chicago, IL, USA). We evaluated the distribution of the data by the Shapiro–Wilk test. Descriptive analyses were used to determine the measures of central tendency and dispersion of the sample to characterize the participants’ profile and the adequacy of the food safety conditions at home. The Kruskal–Wallis test with the Dunn post hoc test was used to compare family income and the Kolmogorov–Smirnov test was used to verify the normal distribution of the number of residents.

We used the chi-square test to evaluate the association between homes’ kitchen classification and participants’ gender. The association between the homes’ kitchen classification and age group of the respondents, schooling, and family income was performed using the Kruskal–Wallis test, with a 5% confidence level. The instrument presents dichotomous variables, so the reliability or internal consistency analysis of the scales was performed by the Kuder–Richardson test (KR-20), where, as in Cronbach’s alpha, correlation values between the analyzed items of 1.00 are considered ideal [22]. Values between 0.6 and 0.8 are considered good quality results for the reliability of the instrument [23].

## 3. Results

The non-normal distribution of the continuous variables of the sample (*p* = 0.000) was observed from the Shapiro–Wilk test. In the present study (*n* = 226 home kitchens), the majority of the interviewees were female (*n* = 146, 64.6%), with completed undergraduate level (*n* = 66, 29.2%) and were aged between 45 and 59 years (*n* = 53, 23.5%, x= 42.37 ± 1.25). The majority of the participants declared the monthly family income to be between 5 and 15 minimum wages (MW) (*n* = 76, 40.0%), followed by 1 to 3 MW (*n* = 55, 28.9%). Households had a mean of 3.38 (±1.48) inhabitants, showing an asymmetric distribution (*p* = 0.000). Table 1 presents the characterization of the research sample according to the socio-demographic characteristics of the interviewees.

According to the first block of the questionnaire (building and electrical facilities), most homes (79%; *n* = 178) were in conformity with the instrument (Table 2). In the second block (equipment, furniture, and utensils), most of the home kitchens (73%; *n* = 165) presented conformity of the evaluated items. The third block, composed of items that evaluated food handlers, presented 75% (*n* = 169) of conformities, classified as medium risk of contamination. The fourth block (raw materials and ingredients) also presented a medium risk of contamination (74%; *n* = 168) in the analyzed homes. The sample can be classified as medium risk environments for food contamination, considering that 46.5% were classified in group 2 (Table 3) in the total of the questionnaire (considering the four blocks).

In the first block, the items that presented the most nonconformities were “waste management” (37% of the home kitchens), followed by “indoor area” (35%), and “urban vector and pest control” (34%). In this block, we observed the presence of pets and disused objects and equipment (indoor area), kitchens with bathroom communication, a waste bin without a lid, and the presence of trash cans on the sink bench (waste management). Most kitchens do not receive disinfection every six months (urban vector), nor have their water reservoir cleaned. In the second block, the items with most nonconformities were “equipment, furniture, and utensils hygiene” (35% of kitchens with nonconformities) and “utensils” (33%) highlighted by pans in poor condition. We observed a low frequency of refrigerator cleaning, a low frequency of changing dish towels, sponges and water filter cartridges, and the presence of steel sponges.

The third block presented the food handlers’ “hygienic habits”, with 29% of the home kitchens with nonconformities, highlighting mainly the lack of adequate hand washing. Finally, in the fourth block, “food storage” presented as the most frequent inadequacy in the evaluated kitchens (41%). This item is also the one with the most present inadequacies considering all the items of the checklist. Households take too much time from preparation until food consumption (delay in food consumption, leaving it at room temperature) as well as food being exposing for a long time at room temperature before refrigerating. Homes demonstrated inadequate storage of perishable food and an elevated risk of cross-contamination when food is stored in a refrigerator or freezer. Partially used ingredients are poorly stored and without identification, and packages are not cleaned before storage.

Most of the home kitchens were classified as group 2, medium risk. However, when evaluating the classification per block, block 4 showed the best classification (group 1), and all others were in group 2. Even though food storage in block 4 had a high number of nonconformities, food origin presented much more conformities, and the block was better classified. The items classification in each block of the houses classified in group 3 is presented in Table S1 (Supplementary File).

We observed that there was no difference by gender (*p* = 0.866), analyzing the characteristics of the food handlers and the surveyed homes. Regarding the age group, there was no statistical difference (*p* = 0.136) in the classification, as well as interviewees’ schooling (*p* = 0.273). Regarding family income, there is a difference by the households (*p* = 0.020), specifically between those who earn from zero to one MW and those who earn from 5 to 15 MW (*p* = 0.017). There was also a significant difference between group zero to one MW and the group above 15 MW (*p* = 0.009), as calculated using the Kruskal–Wallis test with Dunn post hoc test (Table 4).

The reliability of the instrument was confirmed since, from the KR-20 test, the instrument obtained an “acceptable” internal consistency (α = 0.758) [23]. This result suggests that the instrument can measure without error what it proposes; that is, the sanitary safety conditions of food inside domestic kitchens, since the items of each construct can measure it [24].

## 4. Discussion

According to the Brazilian National System of Epidemiological Surveillance of FBD, in the Brazilian Midwest region (location of the Federal District), 55% of the FBD outbreaks occurred from home food ingestion [11,25]. The high number of FBD notifications at home evidences the need to evaluate good food preparation practices in these places, to improve food-handling practices, and to reduce cases of FBDs in the domestic environment [18]. In order to contribute to food safety at home, it is important to establish effective strategies to prevent contamination and to evaluate food preparation. Instruments for the verification of nonconformities in loco that are related to the occurrence of poor hygiene and handling practices may represent an interesting approach to control the production process and provide safe food, considering the absence of studies that investigate possible strategies to prevent contamination [26]. Checklists are used as instruments to assess the safety of food in kitchens. They evaluate the conformity of the items related to food production [27]. Langiano et al. [15] interviewed 1000 individuals using a questionnaire to examine food safety measures. Griglio et al. [28] also used a questionnaire to evaluate food safety at home. In Brazil, we used a validated instrument to evaluate “on-site” the food safety conditions of home kitchens [18]. We tested the reliability of the instrument after applying it in domestic kitchens.

The reliability (internal consistency) of an instrument refers to the measurement of its accuracy using psychometric tests and statistical methods to analyze the data obtained in its application [29]. In the present study, the internal consistency analysis of the checklist was carried out to verify if the items of the instrument were able to measure the conditions of food safety in domestic kitchens. The returned alpha value is considered of good quality for the reliability of the instrument [23] and for the ability to measure food safety conditions in home kitchens [30].

The percentage of cases of FBD due to the preparation of unsafe food practiced at home may be linked to several factors, such as the failure of the food handler, lack of hygiene, improper food storage, poor food preparation conditions, and techniques [14]. In home kitchens, food handlers are usually housewives or housekeepers; however, a study showed that men, young adults, and those with at least some post-secondary education are most likely to contaminate food [9]. Langiano et al. [15] pointed out some incorrect procedures at home more frequently from housewives and high school graduates. Through logistic regression, they presented that high-risk behaviors were influenced by low education level, blue-collar workers, and single status. Unusan [31], evaluating the home kitchens in Turkey, showed that women from 30 to 39 years old were better informed than men. Fisher et al. [32] stated that “*People who prepare food least frequently are the most dangerous cooks*”. In our study, differently from other studies, gender, age, and schooling of the food handler did not interfere in the percentage of conformities. However, family income influenced the risk of food contamination. According to data from the Brazilian Institute of Geography and Statistics (IBGE), the monthly family income of the Federal District was about 2.7 MW in 2018, and it is considered the largest in the country [33]. In our sample, participants with one to three MW comprised 28.9% of the population surveyed, the second most prevalent group. It is crucial to mention that participants earning less than one MW had 16.7% of their homes classified in group 3 (less than 50% of conformities). Fewer earnings can influence the conditions to build houses and their kitchens. Smaller spaces, cheaper materials for construction, fewer available types of equipment, and utensils are factors related to lower income that can influence food safety, especially blocks 1 and 2 of the instrument used in this study. Low income can also influence space for the storage of food.

Although there is no statistical relationship between schooling and the classification of house kitchens, it is worth noting that PDAD 2015 [19] found a statistically significant correlation between the monthly household income and the educational level of the residents of the Federal District. Langiano et al. [15] discussed that food storage, preparation, and cooking are conducted differently with foodborne disease and microbiology knowledge.

Scientific studies conducted in other Brazilian regions suggested that income and the level of schooling of food handlers are factors that influence hygiene knowledge and the adoption of food safety practices in domestic kitchens. However, the authors concluded that the implementation of the government’s food safety education programs can help to improve sanitary food quality in domestic kitchens [34,35]. Families need to be informed about food safety at home and FBD. Not only governments but also health professionals should provide more educational materials to consumers. Redmond and Griffith [36] discussed in their study that the majority of the food safety surveys show a lack of proper information to individuals regarding health risks during home preparation.

In the case of public or private places that sell food, it is necessary to adopt legal administrative measures such as interdiction, application of fines, or written warnings. However, there is no legal regulation for the preparation and storage of food at home, a place most associated with FBD outbreaks in Brazil [37,38]. The leading cause of the incidence of FBD outbreaks at home is the lack of sanitary education and knowledge about food preparation and storage. In our study, the most frequent inadequacies found were incorrect food storage, lack of hygienic habits of food handlers, incorrect waste management, presence of pets, and a lack of urban vector control. However, since we did not evaluate the knowledge of the food handlers involved in food production, it is not possible to evaluate if it this is due to a lack of knowledge or only incorrect action.

Langiano et al. [15] suggested ways to improve food safety that can be used by governments, including the use of brochures at supermarkets with information on microorganisms and printing base concepts of food safety at home cooking on grocery bags. Meanwhile, Unusan [31] suggested media campaigns. Lange et al. [39] studied teachers’ perceptions of food safety at home, and most of the teachers said that schools have the mission to provide knowledge on food safety at home. When evaluating the basic skills at schools, the most performed skills were hand washing, washing up dishes, cleaning of work surfaces, and handling of dishcloths. New creative environments are essential so that students can discuss and problematize food actions at home.

FBD outbreaks resulting from the consumption of contaminated food at home are hardly reported because they involve few people, and the symptoms are mild [4]. Also, our results show that most of the home kitchens were classified as at medium risk of food contamination (group 2) according to the instrument [21]. Studies pointed out that cases on food errors at home are higher than reports with estimations of reaching 95% [36].

Many natural actions occur in home kitchens. They are related to food contamination, such as purses on the kitchen counter, houseplants and pets, the use of the kitchen sink to wash hands, fruits, vegetables, utensils, and pets, and cooked and raw food in contact in the refrigerator. These and other risk factors bring pathogens into the kitchen. During the COVID19 pandemic, media campaigns are encouraging people to pay more attention to common actions performed at home and change this scenario. The population is learning how to wash their hands properly and with more frequency. Also, they need to be more careful with groceries before entering homes, as well as their shoes and clothes. Pets need to be treated with same care to be inside the house as their owners. All this information is provided in educational strategies that may influence in reducing FBD at home. After this pandemic period, changes at home can also influence foods served in church events, school picnics, shared lunches, all of them prepared at home, as highlighted by Behnke et al. [40].

Beard [41] showed that safe food handling was less common in education strategies at schools, and people present limited food preparation experience. Nowadays, this knowledge is more important and emphasizes that we need to discuss, teach, and encourage public policies towards home food safety. Not only do we need to introduce new procedures, but also reinforce them constantly, so actions are recalled and become a routine at home.

## 5. Conclusions

Our results show that the households were classified as medium risk of environmental food contamination (75% of conformities in the total of the instrument). The result of the on-site evaluation confirmed the reliability of the instrument. Unlike other studies with food handlers, gender, age, and schooling did not interfere in the classification of risk of contamination; only the family income influenced the risk of food contamination. The instrument should be used in other cities in Brazil to confirm if these socio variables will have a different influence on in-home food safety. The higher educational level of the FD population is not the same for some regions of the country, and this lack of significance can change. Income was a key element in food safety at home, possibly showing worse results in other cities in Brazil with poorer conditions. Income could be related to more money for better constructions, more space to store foods or more utensils and equipment. Specific education on food safety is necessary, not only formal education in schools. Such findings can contribute positively to the development of actions in health education that help with the adoption of good practices of food manipulation and, consequently, in the reduction in food outbreaks in residences.

## Figures and Tables

**Table 1:** Socio-demographic characterization of the home food handlers in the Federal District, Brazil, 2019.

Variables	*n*	%
Gender		
Female	146	64.6%
Male	80	35.4%
Age group		
18 to 24 years	48	21.2%
25 to 34 years	41	18.1%
35 to 44 years	44	19.5%
45 to 59 years	53	23.5%
60 years and over	40	17.7%
Schooling		
Incomplete elementary school	22	9.7%
Complete Elementary school	12	5.3%
Incomplete High school	19	8.4%
Complete High school	63	27.9%
Incomplete Under-graduation	44	19.5%
Complete Under-graduation	66	29.2%
Family Income		
0 to 1 MW	12	6.3%
1 to 3 MW	55	29.0%
3 to 5 MW	35	18.4%
5 to 15 MW	76	40.0%
Above 15 MW	12	6.3%
Not informed	36	-

MW: Minimum wage = 998 BRL (1 BRL = 5.55 USD).

**Table 2:** Frequency of conformities and nonconformities in home kitchens in the Federal District/Brazil, 2019 (*n* = 226).

Checklist Section	Conformity		Nonconformity	
	*n*	%	*n*	%
Block 1—Construction and Facilities				
Outdoor area	198	88%	28	12%
Indoor area	148	65%	78	35%
Kitchen floor	188	83%	38	17%
Kitchen Ceiling	186	82%	40	18%
Walls and kitchen divisions	186	82%	40	18%
Kitchen door	186	82%	40	18%
Windows and other kitchen openings	189	84%	37	16%
Toilets	163	72%	63	28%
Lightening and kitchen electrical wiring	183	81%	43	19%
Ventilation and acclimatization system of the kitchen	186	82%	40	18%
Urban vector and pest control	150	66%	76	34%
Water supply	180	80%	46	20%
Waste management	143	63%	83	37%
Sanitary sewage	203	90%	23	10%
Block 2—Equipment, Furniture and Kitchen Utensils				
Equipment	182	81%	44	19%
Furniture	179	79%	47	21%
Utensils	152	67%	74	33%
Equipment, furniture and utensils hygiene	148	65%	78	35%
Block 3—Handlers				
Hygienic habits	160	71%	66	29%
Health condition	178	79%	48	21%
Block 4—Food and Feedstock				
Food and feed stock origin	202	89%	24	11%
Food storage	134	59%	92	41%

**Table 3:** Classification of the home kitchens in groups according to conformities to the instrument in the Federal District, Brazil, 2019.

Checklist Section	Classification					
	Group 1 (76–100% of Conformities)		Group 2 (51–75% of Conformities)		Group 3 (0–50% of Conformities)	
	*n*	%	*n*	%	*n*	%
Block 1 (Construction and Facilities)	98	43.4%	119	52.7%	9	4.0%
Block 2 (Equipment, furniture and kitchen utensils)	74	32.7%	124	54.9%	28	12.4%
Block 3 (Handlers)	19	8.4%	113	50.0%	94	41.6%
Block 4 (Food and feedstock)	109	48.2%	64	28.3%	53	23.5%
Total	300	33.2%	420	46.5%	184	20.3%

**Table 4:** Classification of the home kitchens according to the instrument and their relationship with socio-demographic data (gender, age, schooling, and family-wage income) in the Federal District, Brazil. Brasília, DF, 2019.

Variable	Classification					
	Group 1		Group 2		Group 3	
	*n*	%	*n*	%	*n*	%
Gender						
Female	43	29.5%	99	67.8%	4	2.7%
Male	21	26.3%	57	71.3%	2	2.5%
χ^2^ test *p* = 0.866						
Age group						
18 to 24 years	11	22.9%	36	75.0%	1	2.1%
25 to 34 years	12	29.3%	29	70.7%	0	0.0%
35 to 44 years	16	36.4%	26	59.1%	2	4.5%
45 to 59 years	19	35.8%	33	62.3%	1	1.9%
60 years and over	6	15.0%	32	80.0%	2	5.0%
K-W *p* = 0.136						
Schooling						
Incomplete elementary school	4	18.2%	18	81.8%	0	0.0%
Complete Elementary	6	50.0%	6	50.0%	0	0.0%
Incomplete High school	5	26.3%	14	73.7%	0	0.0%
Complete high school	17	27.0%	42	66.7%	4	6.3%
Incomplete Under-graduate	10	22.7%	32	72.7%	2	4.5%
Complete under-graduate	22	33.3%	44	66.7%	0	0.0%
K-W *p* = 0.273						
Family Income						
0 to 1 MW	0	0.0%	10	83.3%	2	16.7%
1 to 3 MW	16	29.1%	38	69.1%	1	1.8%
3 to 5 MW	8	22.9%	26	74.3%	1	2.9%
5 to 15 MW	23	30.3%	52	68.4%	1	1.3%
Above 15 MW	6	50.0%	6	50.0%	0	0.0%
K-W *p* = 0.020						

χ^2^—Chi-square Test. K-W = Kruskal–Wallis test. MW: minimum wage; the homes’ kitchen classification based on the instrument [21]: Group 1—low risk—76 to 100% of the attendance of items; group 2—medium risk—51 to 75% attendance of items; group 3—high risk—0 to 50% of the attendance of items.

## Supplementary Materials

The following are available online at https://www.mdpi.com/1660-4601/17/13/4897/s1, Table S1: Frequency of conformities and nonconformities in home kitchens in the Federal District/Brazil classified as group 3 by the instrument, 2019 (*n* = 06).
