# Supplementary material for: Food Safety Conditions in Home-Kitchens: A Cross-Sectional Study in the Federal District/Brazil
Source: Int J Environ Res Public Health. 2020 Jul 7;17(13):4897. doi: 10.3390/ijerph17134897 (PMC7369695; doi:10.3390/ijerph17134897)
Supplement: Supplementary file 1 [file ijerph-17-04897-s001.pdf]

**Table S1.** Frequency of conformities and nonconformities in home kitchens in the Federal District/Brazil classified as group 3 by the instrument, 2019 ($n$ = 06).

| Checklist Section | Conformity<br>% | Nonconformity<br>% |
|---|---|---|
| Block 1 - Construction and Facilities | | |
| External area free of outbreaks of unhealthiness, garbage accumulation, stagnant water, among others. | 100 | 0 |
| Access roads with adequate sewage system. | 83.3 | 16.7 |
| Kitchen free of obsolete objects or strangers to the environment. | 33.3 | 66.7 |
| Free of the presence of domestic animals. | 50 | 50 |
| Kitchen floor - In good state of cleanliness. | 83.3 | 16.7 |
| Kitchen floor - Cleaned at least once a day. | 66.7 | 33.3 |
| Kitchen floor - Floor in material that facilitates its cleaning (ceramics or similar). | 66.7 | 33.3 |
| Kitchen floor - In suitable state of conservation (free of defects, cracks, cracks, holes, and others). | 33.3 | 66.7 |
| Kitchen Ceiling - With appropriate ceiling material that facilitate cleaning (plaster, pvc, concrete, or similar). | 33.3 | 66.7 |
| Kitchen Ceiling - In suitable state of conservation (free of cracks, humidity, mold, fungus, spider webs, paint peel, and others). | 16.7 | 83.3 |
| Kitchen walls and divisions - In good state of cleanliness. | 33.3 | 66.7 |
| Kitchen walls and divisions - In suitable state of conservation (free of cracks, humidity, paint peel, and others). | 33.3 | 66.7 |
| Kitchen door - In suitable state of conservation (free of cracks, humidity, paint peel, and others). | 0 | 100 |
| Kitchen door - Built with material of easy cleansing. | 50 | 50 |
| Windows and other kitchen openings - In suitable state of conservation (free of cracks, humidity, paint peel, and others). | 33.3 | 66.7 |
| Windows and other kitchen openings - Built with material of easy cleansing. | 16.7 | 83.3 |
| Toilets - Toilet with intact toilet seat and with lid. | 33.3 | 66.7 |
| Toilets - Toilet with running water and connected to the sewage system or septic tank. | 66.7 | 33.3 |
| Toilets - Toilet without direct link to the kitchen area and/or the dining room. | 83.3 | 16.7 |
| Toilets - Toilet with bins with lids and pedal triggering. | 16.7 | 83.3 |
| Lightening and kitchen electrical wiring - Recessed lighting or when external, covered by insulating pipes attached to walls and ceiling. | 0 | 100 |
| Lightening and kitchen electrical wiring - Bulbs and electric switches free of dirt. | 16.7 | 83.3 |

| | | |
|---|---|---|
| Lightening and kitchen electrical wiring - Cleaning of lamps, outlets, and electric switches at least once a month. | 16.7 | 83.3 |
| Ventilation and acclimatization system of the kitchen - Capable of thermal comfort. | 0 | 100 |
| Ventilation and acclimatization system of the kitchen - Kitchen free of fungus, causing no harm to food. | 50 | 50 |
| Urban vector and pest control - Absence of urban vectors and pests or other evidence such as feces, nests, and other. | 16.7 | 83.3 |
| Urban vector and pest control - Disinfecting every six months. | 33.3 | 66.7 |
| Water supply - Water supply system connected to the public grid. | 66.7 | 33.3 |
| Water supply - Proper, protected, covered, and distant from contamination water system. | 66.7 | 33.3 |
| Water supply - Water tank with lid and in good state. | 0 | 100 |
| Water supply - Cleaning of water tank every six months. | 16.7 | 83.3 |
| Waste management - Easy to clean and carry waste bins; in proper state and with appropriate garbage bags. | 16.7 | 83.3 |
| Waste management - Covered waste bins with pedal triggering. | 0 | 100 |
| Waste management - No waste bins over the sink. | 50 | 50 |
| Waste management - Waste stored in appropriate areas. | 66.7 | 33.3 |
| Sanitary sewage - Septic tank and sewage system connected to the public sewage. | 66.7 | 33.3 |
| Block 2 - Equipment, furniture and kitchen utensils | | |
| Equipment - Fridge and stove in an area that permits adequate cleaning. | 33.3 | 66.7 |
| Equipment - Conservation equipment for food (fridges, freezers, and others) in proper functioning. | 66.7 | 33.3 |
| Equipment - Thermal food equipment (stove, oven, and/or microwave) in proper functioning. | 50 | 50 |
| Furniture - From resistant material with proper surface conditions. | 83.3 | 16.7 |
| Furniture - Withdrawing that allows easy cleaning (smooth, without roughness, and chinks). | 50 | 50 |
| Utensils - Size and shape for easy cleaning and in proper state. | 83.3 | 16.7 |
| Utensils - No wooden utensils or other materials of easy contamination. | 50 | 50 |
| Utensils - Pans, pots, and trays in proper state. | 50 | 50 |
| Utensils - Boards, knives, skimmers, and holders in proper state. | 50 | 50 |
| Utensils - Utensils (plates, silverware, bowls) in proper state. | 50 | 50 |
| Equipment, furniture and utensils hygiene: Fridges and freezers in proper state. | 50 | 50 |
| Fridges or freezers cleaned at least once a week. | 33.3 | 66.7 |
| The stove is cleaned when used. | 50 | 50 |

| | | |
|---|---|---|
| Dishcloths in proper and cleaned state. | 50 | 50 |
| Dishrags or table rags in proper state. | 50 | 50 |
| Dishcloths are changed daily. | 50 | 50 |
| Dishrags are changed daily. | 33.3 | 66.7 |
| Cleaning sponges in proper hygiene and state. | 0 | 100 |
| Cleaning sponges changed weekly. | 33.3 | 66.7 |
| Cleaning products approved by the health department. | 33.3 | 66.7 |
| Cleaning products in their original packing and stored in proper location. | 33.3 | 66.7 |
| Equipment, furniture, and kitchen utensils in proper hygiene and state. | 66.7 | 33.3 |
| Water filters changed every six months. | 50 | 50 |
| No sponges from steel or wool. | 66.7 | 33.3 |
| Block 3 - Handlers | | |
| Personal cleaning, good appearance, clean hands, short nails, and unadorned (rings, earrings, bracelets, others). | 50 | 50 |
| Handlers with previous knowledge on hand washing. | 16.7 | 83.3 |
| Absence of skin rash, wound, suppuration; absence of respiratory, eye, and gastric infections. | 16.7 | 83.3 |
| Block 4 - Food and feedstock | | |
| Food and feedstock with labels and packaging according to legislation. | 100 | 0 |
| Milk from a secure source. | 83.3 | 16.7 |
| Cheese from a secure source, packed and labeled. | 83.3 | 16.7 |
| Meat, chicken, or fish from proper establishments. | 83.3 | 16.7 |
| Filtered or boiled water consumption. | 83.3 | 16.7 |
| Semi perishable food stored in adequate and organized area with air circulation and lighting. | 16.7 | 83.3 |
| Food prepared in advance before serving heated again. | 16.7 | 83.3 |
| Fridges and freezers organized in order to avoid cross contamination. | 16.7 | 83.3 |
| Feedstock not used completely are properly stored in clean and closed container. | 16.7 | 83.3 |
| Feedstock not used completely are identified with expiration date. | 16.7 | 83.3 |
| Perishable food stored in adequate temperature. | 16.7 | 83.3 |
| Packages well cleaned before used for fridge or freeze storage. | 16.7 | 83.3 |
| Leftovers stored under refrigeration and with bowls with lids. | 16.7 | 83.3 |
